# Detailed Assessment of the Spatial Distribution of Urban Parks According to Day and Travel Mode Based on Web Mapping API: A Case Study of Main Parks in Wuhan

**DOI:** 10.3390/ijerph15081725

**Published:** 2018-08-11

**Authors:** Qiang Niu, Ye Wang, Yuan Xia, Hao Wu, Xi Tang

**Affiliations:** 1 School of Urban Design, Wuhan University, 299 Bayi Road, Wuchang District, Wuhan 420072, China; niuqiang@whu.edu.cn (Q.N.); 12xiayuan@sina.com (Y.X.); 18672310237@163.com (X.T.); 2 Guangzhou Urban Planning & Design Survey Research Institute, 10-12 Jianshe Road, Yuexiu District, Guangzhou 510060, China; wangyewhu@163.com

**Keywords:** web mapping API, urban parks, assessment of spatial distribution, effective service ratio, accessibility

## Abstract

This article employs two indexes—accessibility and effective service ratio (ESR)—to assess the spatial distribution of urban parks with the consideration of both equity and efficiency. Traditional approaches to calculate these two indexes are often based on the shortest distance to the park within its service radius by network analysis. Such approaches cannot reflect the actual travel behaviors of urban residents and require extensive data collection of road networks and complex parameter setting. To avoid these defects, this study directly acquires travel time data for various travel modes in a specific time period to the park through web mapping API (Application Program Interface), then calculates the accessibility and ESR of urban parks based on these detailed data. This method gets closer to actual park usage situation and avoids the cumbersome process of road network model building. At last, a case study is conducted on the assessment of spatial distribution of main parks in Wuhan, finding that districts with poor park accessibility in Wuhan can be divided into three types: districts with an absence of parks, districts with an insufficiency with parks, and districts separated from parks by traffic. Then, pertinent improvement suggestions are proposed, which provide some bases for decisions on future park planning and construction.

## 1. Introduction

As an important component of the ecosystem of urban greenspaces, urban parks play an important role in public health, urban environment [1,2], and biological diversity [3]. Moreover, it can enhance social ties, create a sense of community, and improve living standards of urban residents [4,5], and it is considered to exert a great influence on the urban economy and real estate market [6,7]. Therefore, offering urban parks which give consideration to both equity and efficiency is of significance for urban sustainable development [8] and physical and psychological health [9] of the residents.

People usually evaluate the spatial distribution of the park with indicators such as per capita park area, green space rate. However, these indicators do not directly reflect the spatial pattern and distribution of parks, and have not taken residents’ actual usage of parks into consideration [10]. For this, scholars proposed evaluation indicators such as accessibility, number of choice opportunities, service area ratio, effective service ratio (ESR), and so on, which can give consideration to both equity and efficiency when evaluating the distribution of parks. The established practice of calculating these indicators is mainly based on the shortest distance to the park within its service radius obtained by network analysis after building road network model. For one thing, these methods are burdensome to build a road network and preset parameters; for another thing, actual travel of residents is somewhat different from the simulated travel. Therefore, this study utilized travel time data by time bucket and travel mode provided by web mapping API (Application Program Interface) of AMap, a leading map company in China, to calculate these two indicators to make the analysis closer to the actual situation and avoid the cumbersome process of constructing road network model in traditional analysis. This method was used to evaluate the spatial distribution of parks in Wuhan, which can provide an effective decision basis for the park planning in the city.

### 1.1. Study on the Spatial Distribution of Parks

A park, as a public facility, has spatial distribution studies that can be traced back to classical location theory. Many classic location theories, such as the location-allocation model [11,12] and the public facility location theory, all emphasize the reasonability of allocation of public facility. Afterwards, Teitz proposed the principle of “efficiency” and “equity” for evaluating the spatial distribution of public facility [11], which gradually became a consensus in academia. McAllister and Lucy further stressed the importance of “equity” [13,14].

To measure the equity of the distribution of parks, many indices are proposed. At the early stage, per capita greenspace area, green space rate, and green coverage rate [14,15] were wildly used, which emphasize the numerical equity of greenspace. Afterwards, research began to focus on spatial equity, and the index of accessibility was valued, which can actually reflect the difficulty of residents to go to parks to some extent [16]. The concept of accessibility was initially proposed by Hansen [17], which is generally defined as the difficulty level of overcoming spatial resistance and expressed with indicators including distance, time and expense. It emphasizes the spatial location of urban parks and the resistance for people to get there [18]. Nowadays, as the equity of different social groups gradually become the focus, on the basis of accessibility, some more elaborate indicators, such as number of choice opportunities [19], supply and demand ratio [20], and so on, are put forward. However, these indicators require complex calculations and are still under development. In sum, accessibility is gradually accepted by the academia, and is often used to reflect equity [4,21]. So we choose accessibility as the main indicator to measure the equity of the spatial distribution of parks.

There are four major methods widely used in accessibility research: the statistical indicator method, the travel distance or expense method, the minimum distance method and gravity model method [16,21,22,23,24]. The travel distance or expense method is the most widely used among them, which reflects accessibility by calculating accumulative resistance (distance, time, and expense) between parks and residents. With different understandings and applications of the resistance, this method can be divided into the simple buffer method [21], the cost weighted distance method [25,26], the network analysis method [21,27], and the two-step floating catchment area (2SFCA) method [28,29,30].

In addition to the equity of the distribution of parks, efficiency is also an important issue, which refers to the actual service effect per unit area of urban parks, namely the supply capacity of facilities mapping on space [31]. At present, some indicators are proposed to measure the service distribution efficiency of urban parks, such as the service coverage ratio [32], service area ratio [33], effective service ratio (ESR) [34]. The meanings of these indicators are basically similar, which are the proportion of the service area of parks in the area of the analysis region. But there are still some differences in the area calculation of the analysis region: service coverage ratio takes the entire area of the analysis region into the calculation, it can be understood as gross efficiency; service area ratio counts the non-park area of the entire analysis region; ESR improves further, which only counts the actual area in use of the parks, and thus removes the area of park and water in the entire analysis region, it can be understood as net efficiency, and is relatively more accurate. Besides, the method of calculating the service area is also constantly improving. Service area is the service range of a public facility which is equivalent to the accessibility to a public facility such as a park or school that supplies service via traffic networks [16]. Scholars of early stages usually use simple buffering which involves drawing lines around parks at a given distance [35], it focuses on the linear distance from parks rather than considering citizens’ actual routes to them, however, it is simple to calculate. In recent years, the method of using simulated travel time or distance by network analysis rather than the straight line distance to calculate service area becomes more and more popular. Although the accuracy is greatly improved, the workload of calculation is heavier, too. With the rise of big data, the method of calculating service area according to relative actual travel route and travel time provided by web mapping API has been put forward [36], but has been little used in analyzing the spatial distribution of parks. In addition to these area-based indicators, there are indicators based on the number of accessible parks such as service overlap degree [32], which is the number of urban public green spaces serving for a specific residential unit, and reflects the selectivity of using parks, or the waste of resources. There are also indicators based on the service population, such as the service population ratio [33], which refers to the ratio of the population served in the service area to the total population of the analysis region. It takes into account both the spatial distribution of the population and the spatial extent of the service, so it’s relatively more accurate, but requires the support of detailed population distribution data. Under the premise of limited data sources, we choose to use ESR to evaluate the efficiency. This indicator is not only easy to obtain, but also can better reflect the guiding significance of park planning from the spatial perspective.

ESR refers to the percentage of the service area of parks in a research unit (minus the area of park and water) [34]:

(1) $$ESR=\frac{S_{effective\;service}}{S_{research\;unit}-S_{park}-S_{water}}$$

where *S_effective service_* refers to effective service scope (ESS) of a park, which is the surrounding area of a park within its service radius with the area of park and waters excluded. Governments around the world have set clear standards for the service radius (area) of park. For example, it is stipulated by the British government that the minimum areas of parks and the maximum distances from greenspaces to homes are 2 hm^2^ and 300 m, 20 hm^2^ and 2 km, 100 hm^2^ and 5 km, 500 hm^2^ and 10 km respectively [4]. In this article, we estimated the ESS according to residents’ time expense to go to the park, for example, the area within 20 min’ walk to get to is regarded as the walking ESS of parks. This can better reflect the real situation and feelings of the residents about going to the park than service radius does. *S_research unit_*is the total area of a research unit, *S_park_* is the area of the park within the research unit, and *S_water_* is the area of the water within the research unit. The denominator means the actual area in use of the parks.

### 1.2. Web Mapping API

**Figure 1:**
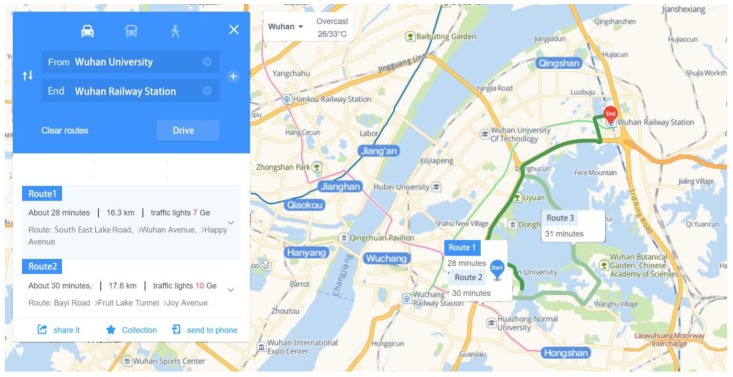
Recommended travel routes of AMap.

At present, mainstream map service providers such as Google Maps, Bing Maps, and AMap provide users with the shortest route, the transportation recommended, and the time prediction from one point to another. Taking AMap, a Chinese local map service provider, as an example, it provides users with recommended travel routes for different travel modes and time estimates (Figure 1). These providers also provide an interface (web mapping API) for researchers to acquire these data automatically by calling corresponding API, which proffers to us a new approach to measure accessibility. Related studies proved these map services are based on a big amount of real historical and real-time data, use scientific computing methods and offer accurate, reliable results [36,37,38,39]. Web mapping API has been preliminarily used in the studies of OD (Origin-Destination) analysis [36], measurement of walking accessibility [37], accessibility [38] of business centers, and so forth. Compared with network analysis, the use of web mapping API has a number of advantages, such as no need to build a road network, using the latest road network and reflecting real-time traffic situation [39]. Therefore, this article selected web mapping API to analyze accessibility and ESR of parks.

This experiment selected web mapping API data provided by AMap for analysis, for it possesses large amount of high-precision real traffic data and actively applies the latest AI (Artificial Intelligence) technology among various technologies to calculation, and as a result, the accuracy of its forecasts of the travel time is ensured.

## 2. Materials and Methods

### 2.1. Overview of the Study Area

This study selected Wuhan City, China, in which to conduct the case study. Wuhan is newly-selected national central city in China. As an economic, political and cultural center of central China, Wuhan has a resident population of 10.914 million in 2017. Its topography is composed of large rivers and huge lakes, thus creates a distinctive feature of the spatial distribution of parks in Wuhan. The parks in Wuhan city are mainly built on the mountain and the water, many of them are big. In recent years, park increasingly becomes a destination for residents to stroll about or have a rest, as the gradually perfecting of urban transportation system. This study selected the most densely populated urban area in Wuhan as the study area, whose total area is 975.12 km^2^, and chose its main parks as the study objects.

### 2.2. Data

**Figure 2:**
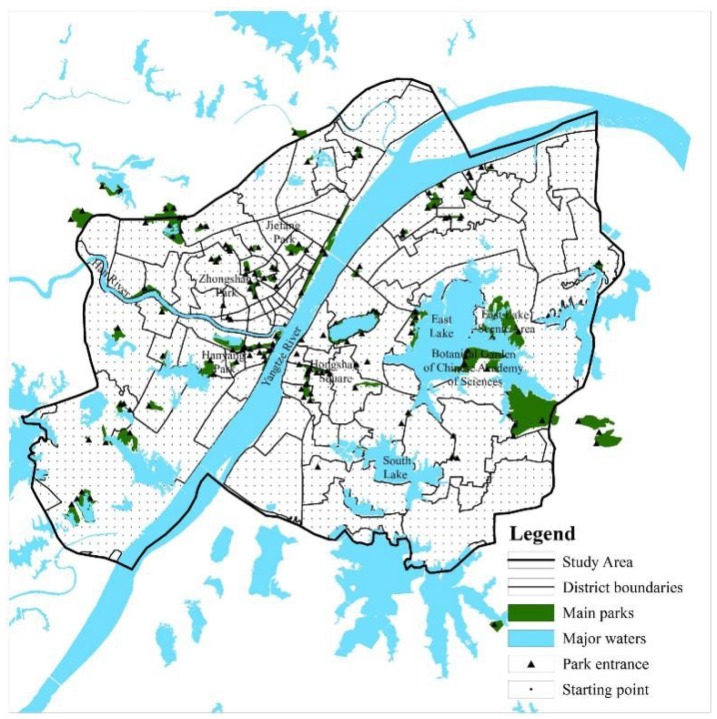
The distribution of main parks in Wuhan.

This study uses administrative regions of district level in Wuhan as the basic evaluation units of ESR calculating, for each district has a considerable proportion of residential land and residential population, and it is the responsibility of the government in this level to construct parks. District data came from the administrative map of Wuhan. According to this map, district boundaries were drawn on ArcGIS and 92 districts were obtained as our basic evaluation units (Figure 2).

Data of parks was sourced from Catalogue of Main Parks in Wuhan. Seventy-eight main parks within or near downtown Wuhan were selected according to their level, size, and actual construction condition. These parks include specialized parks like East Lake Scenic Area and Botanical Garden of Chinese Academy of Science; city-level parks such as Zhongshan Park and Jiefang Park; and large district-level parks like Hongshan Square and Hanyang Park.

Travel starting point data for the calculation of accessibility are intersection points of the 500 m × 500 m grid demarcated by ArcGIS in the study area. Additionally, 2238 starting points were obtained after removing parks and inaccessible areas like waters.

Travel destinations of accessibility calculation are the entrances of each park. We used the actual survey and online inquiry to find out the entrances and obtained 159 destination points and their coordinates.

### 2.3. Methods

This article uses time costs calculating method provided by AMap’s web mapping route planning API to measure the accessibility and ESR of parks, and the specific steps are detailed below.

#### 2.3.1. Acquisition of Minimum Time Cost

AMap’s route planning API provides users with some recommended routes from one point to another, which covers the selection of different modes of transport and an estimation of travel time, and it can export these data as required (Table 1). This study used the API to batch obtain the time expense from 2238 starting points to 159 park entrances in the study area at the same time. For each starting point, the minimum time spent among all the recommended routes to 159 parks is treated as its accessibility to parks.

In view of habits of people using parks in their daily life, we subdivide accessibility and ESR into three travel modes and two time buckets:

Three travel modes: walking, car, and public transport. Related data on the three travel modes were recorded respectively. Car and public transport both choose the fastest route, and public transport includes rail transit like metro.

Two time buckets: workdays and weekends. As traffic condition has little influence on walking, only data on going out by car and public transport at the two buckets were recorded. For workdays, data on 7 p.m. from Monday to Friday from 15 to 19 May in 2017 were recorded, and their mean value was selected as the minimum cost; for weekends, data on 10 a.m. and 7 p.m. from Saturday to Sunday of the three weeks from 8 to 28 May of 2017 were recorded, and their mean value was selected as the minimum cost. 

#### 2.3.2. Evaluation of the Accessibility of Parks

Minimum time cost for each starting point to go to the park was taken as an indicator to measure accessibility. Based on minimum costs of each starting point acquired in the previous step, the study used spatial interpolation to generate the grid map of park accessibility in the whole study area. Furthermore, walking accessibility is divided into four ranks: within 10 min, 10–20 min, 20–30 min, and over 30 min; car and public transport accessibility is divided into four ranks: within 15 min, 15–30 min, 30–60 min, and over 60 min. 

#### 2.3.3. Evaluation of ESR of Parks

Firstly, we determined the criterion to calculate the ESS of parks for each travel mode. We let residents choose the acceptable time expense on going to parks in the aforesaid accessibility ranks. Through 498 questionnaires, it turned out that 72% of respondents chose within 20 min by walking, 65% chose within 30 min by public transport, and 63% chose within 15 min by car. Thus, we regard the scope around the park within 20 min’ walk or 30 min’ drive or 15 min on public transport as ESS of parks.

Then, based on the map of park accessibility, we summarized park ESS of each travel mode for the whole study area and its 92 districts, and calculate park ESP according to the Equation (1) respectively. Taking the calculating of Walking ESR of Zhongnan District (District NO. 19) as an example, *S_research unit_* of Zhongnan District is 11.1585 km^2^; there is one park with an area of 0.4495 km^2^ in it and three parks nearby, which indicate that its *S_park_* is 0.4495 km^2^; according to the aforementioned rules, its *S_effective service_* is the area around these four park within 20 min’ walk in the district, which is 4.6546 km^2^; and there is no water area in the district, so the *S_water_* is 0 km^2^. Taking these data into Equation (1), we get an ESR of 0.4346.

## 3. Results

### 3.1. Walking-Based Evaluation Results

Walking is the most fundamental transport mode with numerous advantages for it is economical, convenient, low carbon, environmentally friendly, free and flexible, and it is the first choice for urban residents to go to the park.

**Figure 3:**
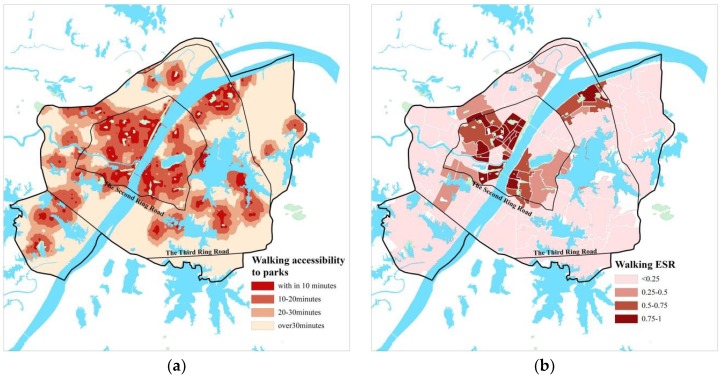
(**a**) Spatial distribution of walking accessibility to parks; (**b**) Spatial distribution of walking ESR.

According to the calculation results, in terms of the whole study area, its walking accessibility to main parks in Wuhan is poor (Table 2, Figure 3a), the effective service scope (ESS) by walk (within 20 min’ walk) covers an area of 214.2 km^2^, with an effective service ratio (ESR) of 28.34%. In terms of the districts (Figure 3b), there are obvious spatial differences of the walking ESR among them: the districts within the Second Ring Road are of a relatively higher walking ESR, and their ESR decreases from the core to the outskirts.

### 3.2. Car Traffic-Based Evaluation Results

According to statistical data, the motor vehicle number of Wuhan has increased to 2.7 million in 2017. The growing number of motor vehicles and the emergence of online car-hailing have made car travel a major supplement for urban residents to go to parks.

**Figure 4:**
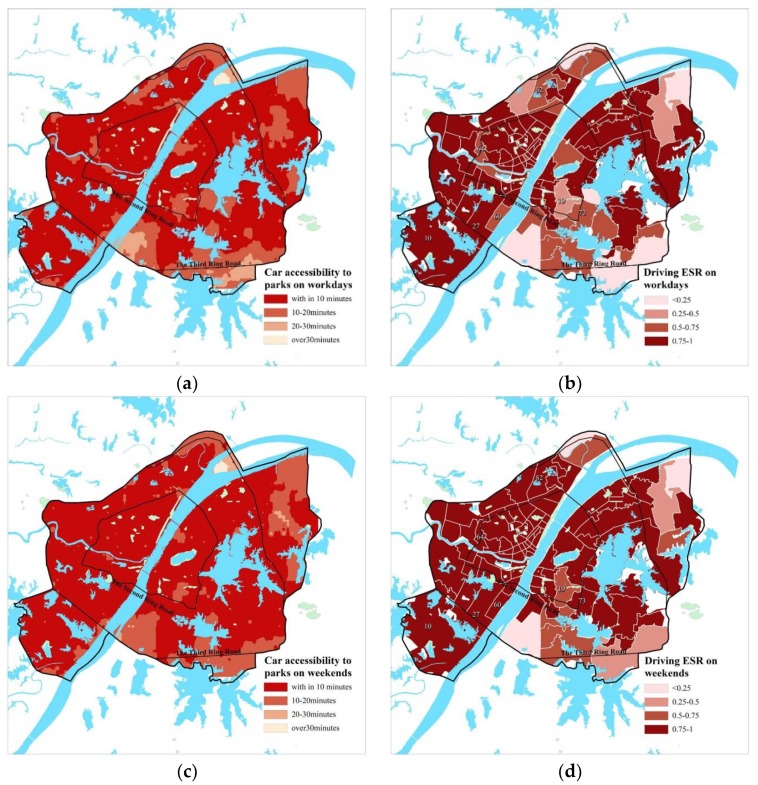
(**a**) Spatial distribution of car accessibility on workdays; (**b**) Spatial distribution of car ESR on workdays; (**c**) Spatial distribution of car accessibility on weekends; (**d**) Spatial distribution of car ESR on weekends.

According to the calculation results, in terms of the whole study area (Table 3, Figure 4a,c), car accessibility to main parks in Wuhan is relatively high, yet they are greatly affected by traffic conditions. The time urban residents usually use the parks is around 7 p.m. on workdays, which overlaps with evening peak, hence, car travel-based park service level varies greatly between weekdays and weekends. Its ESS by car (within 15 min’ drive) on workdays covers an area of 550.7 km^2^, with an ESR of 72.85%; while on weekends, its ESS significantly rises to an area of 597.8 km^2^, with an ESR of 79.07%. This is mainly due to the relatively smooth traffic on the weekend, which also proves the necessity to do assessment according to day.

In terms of the districts (Figure 4b,d), outskirt districts may have a higher car accessibility and ESR than core districts. Some outskirt districts, such as Jiangdi (District NO. 27), Zhoutou (NO. 60) and Zhuankou (NO. 10) Districts, have good traffic conditions and abundant park resources, hence their park accessibility and ESR are significantly improved by car; on the contrary, some core districts, such as Zongguan (NO. 64), Houhu (NO. 82), Zhongnan (NO. 19) and Luonan (NO. 73) Districts, although abound in parks, are greatly affected by traffic jam, and have relatively low ESR on workdays. But over the weekend, with the improvement of traffic conditions, it can be seen from Figure 4c that the ESR of these areas get an improvement of about one grade.

### 3.3. Public Transport-Based Evaluation Results

Public transport has many advantages for it is intensive, efficient, low-carbon and environmentally friendly, and it is a major tool to relieve traffic jams, transform traffic modes, and improve travelling quality for residents. In recent years, Wuhan energetically developed public transportation with a focus on the metro system that seven metro lines are in operation and another seven lines will be buildup by 2025 with a total construction mileage of 423 km. The growth of public transportation can relieve urban traffic pressure and improve the accessibility of Wuhan parks.

**Figure 5:**
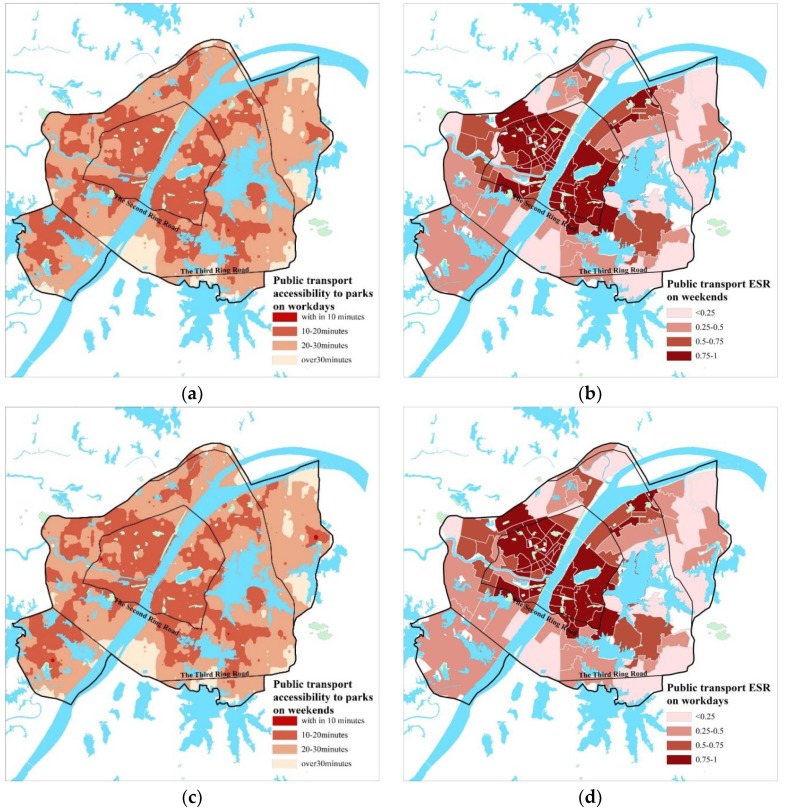
(**a**) Spatial distribution of public transport accessibility on workdays; (**b**)Spatial distribution of public transport ESR on workdays; (**c**) Spatial distribution of public transport accessibility on weekdays; (**d**) Spatial distribution of public transport ESR on weekends.

According to the calculation results, in terms of the whole study area (Table 4, Figure 5a,c), public transport accessibility to main parks in Wuhan is unsatisfactory, which shows that although public transportation in Wuhan has made progress in recent years, the transit system is still not efficient enough, thus the improvement of the park accessibility that public transit brings is limited, but it’s believed to progress in the future after the buildup of the rail transit system. At present, ESS of public transport on workdays (within 30 min on public transport) covers an area of 326.3 km^2^, with an ESR of 43.17%; on weekends, its ESS cover an area of 331.4 km^2^, with an ESR of 43.67%. In terms of the districts (Figure 5b,d), ESR varies greatly among districts and shows the characteristic of progressive decreases from the core to the outskirts of the city. However, ESR is almost indistinctive between workdays and weekends. This is mainly due to the rail transit network. The public travel based on the subway avoids the influence of the ground traffic conditions, making the travel time by public transport is not much different between workdays and weekends.

### 3.4. Typical District Types with Poor Park Accessibility and Improvement Suggestions

**Figure 6:**
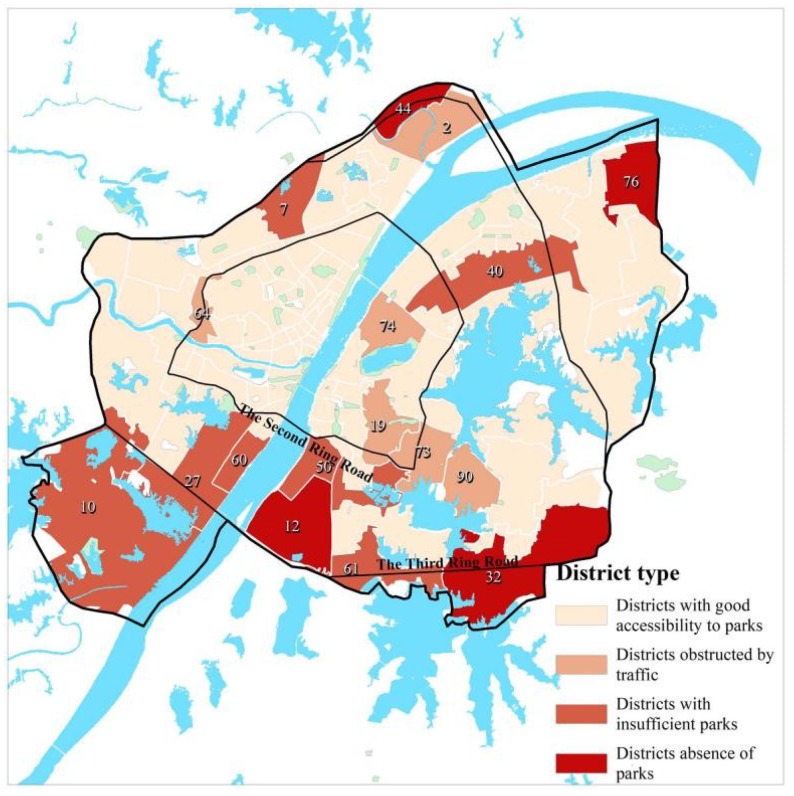
Typical district classification.

By combining the above three kinds of calculation results based on different travel modes with field investigation, districts with poor park accessibility in the study area can be divided into three types: districts absence of parks, districts insufficiency with parks and districts separated from parks by traffic (Table 5, Figure 6).

Districts absence of parks are primarily located on the outskirts. As the result of the rapid urban expansion, such districts generally are the new field for urban development; these have fewer residents, incomplete infrastructures and facilities, and are absent of parks. Thus, their future construction needs to strictly abide by urban public green space planning, stick to urban green and blue lines, and be in line with local conditions.

Districts insufficiency with parks are largely concentrated in the area outside the Second Ring Road. Such districts are the main battlefield for current urban construction and have built some parks, but the number and spatial distribution of parks are unbalanced. Although their walking accessibility to parks is poor, their driving accessibility is good which make good compensation. It is suggested such areas should add a certain number of parks, strengthen their linkage with public transportation, and improve their service level.

Districts separated from parks by traffic are mainly gathered in the core circle. Such districts are mostly located in the old town. Although they are not far away from city parks in terms of linear distance, yet large traffic flow and low traffic capacity as well as hindrances such as river, railway, and overpass make it difficult to get access to the parks despite of proximity. Hence, it is suggested to build more foot-bridges or pedestrian underpasses to traverse traffic barrier in future; meanwhile, it is advisable to make up for the deficiencies of parks in the old town by speeding up the planning and construction of community-level greenspaces and other means.

## 4. Discussion

This study calculated the accessibility and service area ratio on the basis of web mapping API data provided by AMap to evaluate the spatial distribution of the parks in Wuhan. Compared with traditional network analysis, using web mapping API data has the following advantages:

1. Accurate and real-time road network model. Their urban road network is already very accurate, the accuracy of which can generally reach the residential road-level in large cities. Additionally, these web maps can provide researchers with the latest road data. Taking Google Maps as an example, its update cycle is usually twice per month;

2. Accurately reflect the actual traffic condition. With the popularization of smartphones and their locating function, map service providers capture enormous location data generated when users are using such map apps in real time, thus accurately reflecting the actual traffic conditions in real time;

3. Detailed travel time prediction by time bucket and travel mode. The estimate is based on the mobile position big data collected from a large number of travelers in real time. It can reflect the traffic conditions such as road congestion and waiting at the intersection in real time, and even indirectly reflect the influence of road width, undulation, and streetscape on traffic by travelers’ moving speed. So, web mapping API can predict the time required for multiple travel modes and offer multiple route plans more accurately.

4. Easy to access, ready to use. Currently, web map service providers generally provide abundant data access interfaces, which makes travel data can be easily acquired through these APIs.

However, there are some limitations of using the web mapping API directly:

1. The accuracy of the estimated travel data is relative to the traditional simulation method, and there are a certain range of deviations comparing with the actual situation, and it mainly depends on the web map providers we choose, the number of mobile position data that provider collects, the modeling accuracy of roads, and even the weather (bad weather can lead to mobile phone positioning failure or larger deviation), etc. This will directly lead to deviations in the evaluation results. To reduce this uncertainty, we recommend using the data from domestic web map providers, for they usually have more comprehensive data and larger user base, which is the basis of accuracy. For example, AMAP and Baidu Map are more recommended in China, while in Europe, Google Map is more suitable. In addition, in order to reduce the random error, the method of repeated sampling can be used. For example, in the calculation of the park accessibility and ESR on working days in our paper, we collected data of five days from 15–19 May in 2017, and take the average as the evaluating data. In addition, data can be collected from multiple web map providers at the same time, which can improve the reliability of the data to some extent.

2. At present, the prediction of walking time and route by these web map providers is based on the large probability value speculated from the travel data of a large number of travelers. Special populations such as pregnant women, disabled people, and the elderly have not been considered yet, so it is difficult to carry out detailed research on these sub-populations. However, such particular route planning does exist, such as route plan for the disabled which should be based on barrier-free road, so it is believed that web map providers will supplement their research in the future and provide relevant APIs.

## 5. Conclusions

Parks are significant for improving city quality and living standards of residents, and the spatial distribution of a park determines its service level. This study utilized web mapping API data to evaluate the spatial distribution of park, dispensed with the complicated process of building road network data and calibrating parameters in traditional analysis, and considered actual usage of urban public parks with different days and modes of transport under the support of location big data owned by web map service providers. After the evaluation of spatial distribution of large parks in Wuhan City through day and travel mode, some polarization spatial distribution features were found: the inner circle has more parks with overall good accessibility and high service level, but some districts are hard to get access to public parks due to traffic obstructions; while the outskirts have less parks with poor walk accessibility, yet driving or public transit can make up for it since there are less traffic obstructions. Besides, the car accessibility and ESR rise on the weekends due to the release of traffic congestion, but public transport accessibility and ESR do not show such features. These conclusions are of practical significance for future targeted planning and construction of parks.

This study was an attempt to evaluate the spatial distribution of urban parks with the support of the location big data platforms of web maps. This method is simple, easy to operate, and able to be applied in towns, cities and metropolises as long as the web mapping API data cover these areas. Besides, the accessibility calculating method is applicable for schools, hospitals and other public service facilities, and ESR is also essentially applicable after the slight adjustment of Equation (1) to make the denominator equals to the actual area in use of the facilities. There are still some limitations in this study: the 500 m × 500 m dot matrix division of main urban area has an impact on the accuracy of accessibility calculation. Generally, the denser the points, the higher the accuracy will be, but the larger amount of calculation will take. In addition, the evaluation of the distribution of parks in this paper mainly considers the supply level of the park from the view of distribution, and has not combined the public’s demand for the park. This may lead to an overestimated ESR in sparsely populated unit, or an underestimated ESR in densely populated unit. The solution is to narrow the evaluation unit to the community level, or to improve the Equation (1), and take the ratio of the resident population in the ESS to the total population in the unit as ESR. As these methods require more detailed data support, we will further improve them in the follow-up study. Besides, the study doesn’t consider the equity of the distribution of parks for the special populations such as the disabled and the elderly, we will carry out a more detailed sub-population evaluation after the route planning APIs for special populations are available.

## Figures and Tables

**Table 1:** Main outputs of AMap’s walking route planning API.

Name				Type	Meaning
count				numerical value	total number of returned routes
route				object	routes information list
	origin			numerical value	coordinate of starting point
	destination			numerical value	coordinate of end point
	paths			array	paths information list
		distance		numerical value	walking distance of the path
		duration		numerical value	estimated walking time
		steps		array	walking steps list
			step	object	each section of walking path, include their road names, distances, time expense and coordinate points

**Table 2:** Walking accessibility scale.

Accessibility	Area (ha.)	Percentage
within 10 min	4694.80	6.21%
10–20 min	16,726.95	22.13%
20–30 min	15,601.55	20.64%
more than 30 min	38,570.33	51.02%
total	75,593.63	100.00%

**Table 3:** Car accessibility scale.

Accessibility	Area on Workday (ha.)	Percentage	Area on Weekend (ha.)	Percentage
within 15 min	55,073.03	72.85%	59,775.13%	79.07%
15–30 min	17,452.13	23.09%	14,779.96	19.55%
30–60 min	2624.19	3.47%	649.82	0.86%
more than 60 min	444.28	0.59%	388.71	0.51%
total	75,593.63	100%	75,593.63	100%

**Table 4:** Public transport accessibility scale.

Accessibility	Area on Workday (ha.)	Percentage	Area on Weekend (ha.)	Percentage
within 15 min	5952.56	7.87%	6191.76	8.19%
15–30 min	26,681.32	35.30%	26,817.50	35.48%
30–60 min	35,638.52	47.14%	35,359.93	46.78%
more than 60 min	7321.23	9.68%	7224.44	9.56%
total	75,593.63	100.00%	75,593.63	100.00%

**Table 5:** Typical district classification.

Types	Districts
Districts absence of parks	Zhangjiawan (NO. 12), Fozuling (NO. 32), Bajifu (NO. 76), Shekou (NO. 44)
Districts insufficiency with parks	Zhuankou (NO. 10), Jiangdi (NO. 27), Zhoutou (NO. 60), Heping (NO. 40), Bashazhou (NO. 50), Hongshan (NO. 61), Tazihu (NO. 7)
Districts separated from parks by traffic	Chenjiaji (NO. 2), Zongguan (NO. 64), Zhongnan (NO. 19), Luonan (NO. 73), Zhuodaoquan (NO. 90), Xujiapeng (NO. 74)

## References

[1] Escobedo F.J., Kroeger T., Wagner J.E. (2011). Urban forests and pollution mitigation: Analyzing ecosystem services and disservices. Environ. Pollut..

[2] Gadi V.K., Tang Y.R., Das A., Monga C., Garg A., Berretta C., Sahoo L. (2017). Spatial and temporal variation of hydraulic conductivity and vegetation growth in green infrastructures using infiltrometer and visual technique. Catena.

[3] Kowarik I. (2011). Novel urban ecosystems, biodiversity, and conservation. Environ. Pollut..

[4] Comber A., Brunsdon C., Green E. (2008). Using a GIS-based network analysis to determine urban greenspace accessibility for different ethnic and religious groups. Landsc. Urban Plan..

[5] Wendel H.E.W., Zarger R.K., Mihelcic J.R. (2012). Accessibility and usability: Green space preferences, perceptions, and barriers in a rapidly urbanizing city in Latin America. Landsc. Urban Plan..

[6] Correll M.R., Lillydahl J.H., Singell L.D. (1978). The Effects of Greenbelts on Residential Property Values: Some Findings on the Political Economy of Open Space. Land Econ..

[7] Votsis A. (2017). Planning for green infrastructure: The spatial effects of parks, forests, and fields on Helsinki’s apartment prices. Ecol. Econ..

[8] Grădinaru S.R., Hersperger A.M. (2018). Green infrastructure in strategic spatial plans: Evidence from European urban regions. Urban For. Urban Green..

[9] Richardson E.A., Pearce J., Mitchell R., Kingham S. (2013). Role of physical activity in the relationship between urban green space and health. Public Health.

[10] Wolch J.R., Byrne J., Newell J.P. (2014). Urban green space, public health, and environmental justice: The challenge of making cities ‘just green enough’. Landsc. Urban Plan..

[11] Teitz M.B. (1968). Toward a theory of urban public facility location. Pap. Reg. Sci..

[12] Bach L. (1980). Locational models for systems of private and public facilities based on concepts of accessibility and access opportunity. Environ. Plan. A.

[13] Mcallister D.M. (1976). Equity and Efficiency in Public Facility Location. Geogr. Anal..

[14] Lucy W. (1981). Equity and Planning For Local Services. J. Am. Plan. Assoc..

[15] Lineberry R. (1977). Equality and Urban Policy: The Distribution of Municipal Public Service.

[16] Talen E., Anserine L. (1998). Assessing Spatial Equity: An evaluation of Measures of Accessibility to Public Playgrounds. Environ. Plan. A.

[17] Hansen W.G. (1959). How Accessibility Shapes Land Use. J. Am. Inst. Plann..

[18] Pirie G.H. (1979). Measuring accessibility: A review and proposal. Environ. Plan. A.

[19] Qiuxiao C., Yan H., Shuang W. (2016). Assessment of Accessibility to Urban Parks in Shaoxing City from the Perspective of Opportunity. Geogr. Sci..

[20] Haiwei Y., Jiangang X. (2009). Spatial Accessibility and Equity of Parks in Shanghai. Urban Stud..

[21] Nicholls S., Shafer C.S. (2001). Measuring accessibility and equity in a local park system: The utility of geospatial technologies to park and recreation professionals. J. Park Recreat. Admi..

[22] Xiaoma L., Changfu L. (2009). Accessibility and service of Shenyang’s urban parks by network analysis. Acta Ecol. Sin..

[23] Hodgart R.L. (1978). Optimizing Access to Public Services. Prog. Hum. Geogr..

[24] Yin H.-W., Kong F.-H., Zong Y.-G. (2008). Accessibility and equity assessment on urban green space. Acta Ecol. Sin..

[25] Yu K. (1999). Landscape Accessibility as a Measurement of the Function of Urban Green System. City Plan. Rev..

[26] Yin H.W., Kong F.H. (2006). Accessibiliy Analysis of Urban Green Space in Jinan. J. Plant. Ecol..

[27] Yin H., Jiangang X. (2009). Spatial Accessibility and Equity of Parks in Shanghai. Urban Stud..

[28] Radke J., Mu L. (2000). Spatial Decompositions, Modeling and Mapping Service Regions to Predict Access to Social Programs. Geogr. Inf. Sci..

[29] Chunliang X., Rui G., Qi W. (2014). Evaluation of green space accessibility of Shenyang using Gaussian based 2-step floating catchment area method. Prog. Geogr..

[30] Dai D. (2011). Racial/ethnic and socioeconomic disparities in urban green space accessibility: Where to intervene?. Landsc. Urban Plan..

[31] Fang M. (2010). Research on Evaluation Method of Service Level of Public Facilities Space-Take the Wuhan Public Library as an example. Planners.

[32] Yu B.L., Hu Z.M., Wu J.P., Qian J., Hu C.L., Tan W.Q., GUO Z.Y. (2013). Analyzing The Social Service Function of Urban Public Green Space For The Residential Disdricts Quantitatively—A Case Study of The Central Area of Shanghai, China. Resour. Environ. Yangtze Basin.

[33] Kyushik O., Seunghyun J. (2007). Assessing the spatial distribution of urban parks using GIS. Landsc. Urban Plan..

[34] Xiao H., Yuan Q., Xu H. (2009). Green Space Distribution Based on Accessibility and Serving Area. Planners.

[35] Ahn T.M., Choi H.S., Kim I.H., Cho H.J. (1991). A study on the method of measuring accessibility to urban open spaces. Landsc. Archit..

[36] Karimi H.A. (2015). A comparative analysis of routes generated by Web Mapping APIs. Cartogr. Geogr. Inf. Sci..

[37] Zhou X., Kim J. (2013). Social disparities in tree canopy and park accessibility: A case study of six cities in Illinois using GIS and remote sensing. Urban For. Urban Green..

[38] Feilong H., Shijun W., Dongcan X., Tingting Y., Zhangxian F. (2017). Space-Time Accessibility of Commercial Centers in Changchun Urban Area Based on Internet Map Service. Econ. Geogr..

[39] Fahui W., Yanqing X. (2011). Estimating OD travel time matrix by Google Maps API: Implementation, advantages, and implications. Geogr. Inf. Sci..

